# The relationship between vacuolation and initiation of PCD in rice (*Oryza sativa*) aleurone cells

**DOI:** 10.1038/srep41245

**Published:** 2017-01-24

**Authors:** Yan Zheng, Heting Zhang, Xiaojiang Deng, Jing Liu, Huiping Chen

**Affiliations:** 1 Key Laboratory of Protection and Development Utilization of Tropical Crop Germplasm Resources (Hainan University), Ministry of Education, Haikou 570228, China; 2 College of Horticulture and Landscape Architecture, Hainan University, Haikou 570228, China

## Abstract

Vacuole fusion is a necessary process for the establishment of a large central vacuole, which is the central location of various hydrolytic enzymes and other factors involved in death at the beginning of plant programmed cell death (PCD). In our report, the fusion of vacuoles has been presented in two ways: i) small vacuoles coalesce to form larger vacuoles through membrane fusion, and ii) larger vacuoles combine with small vacuoles when small vacuoles embed into larger vacuoles. Regardless of how fusion occurs, a large central vacuole is formed in rice (*Oryza sativa*) aleurone cells. Along with the development of vacuolation, the rupture of the large central vacuole leads to the loss of the intact plasma membrane and the degradation of the nucleus, resulting in cell death. Stabilizing or disrupting the structure of actin filaments (AFs) inhibits or promotes the fusion of vacuoles, which delays or induces PCD. In addition, the inhibitors of the vacuolar processing enzyme (VPE) and cathepsin B (CathB) block the occurrence of the large central vacuole and delay the progression of PCD in rice aleurone layers. Overall, our findings provide further evidence for the rupture of the large central vacuole triggering the PCD in aleruone layers.

As cereal grain matures, the surface cells of the endosperm differentiate into the aleurone cells^1^, and the mature aleurone cells fill with organelles, especially protein storage vacuoles (PSVs). The aleurone layers of most cereal seeds only have one layer of cells, but barley and rice seeds can have three or four layers of cells. During cereal seed germination, the aleurone cells synthesize and secrete hydrolytic enzymes into the starch endosperm to degrade stored substances into small molecular substances for seed germination and early seedling growth, and then these aleurone cells die; this is defined as programmed cell death (PCD). Thus, the PCD of aleurone cells is an essential process for the successful completion of cereal seed germination, which is the vacuole-mediated cell death associated with the destruction of the vacuole^2^,^3^.

The vacuole is the largest organelle in plants. There are two types of vacuoles in plant cells, i.e., PSVs and lytic vacuoles (LVs); the large central vacuole is a special type of LV^4^,^5^,^6^. PSVs mainly accumulate all kinds of proteins, such as defense proteins and storage proteins, as well as carbohydrates, etc. and their pH value is close to neutral. By contrast, LVs contain numerous hydrolytic enzymes used in the degradation of intracellular unwanted material, and their roles are similar to yeast vacuoles and animal lysosomes in maintaining acidic conditions. The two types of vacuoles co-exist in the same cells for a short time and may also be present in cells at different stages of development^7^,^8^,^9^. The complex fusion of PSV membranes and the changes in the vacuole content lead to the establishment of LVs^10^. In recent years, the origin of plant vacuoles has been studied^11^,^12^; however, the formation of the LV type remains unclear. Paris *et al*.^7^suggested that young root tips contain PSV and LV types, which fused into a large central vacuole. However, Olbrich *et al*.^9^ showed that root cells initially only contain PSVs. When vetch (*Vicia sativa*) seeds were germinated for 2 to 3 d, storage proteins begin to be mobilized^13^,^14^. PSVs gradually become acidic and fill with active hydrolytic enzymes, along with structural changes in the PSVs, and the vacuole starts the transformation from the PSV type to the LV type^15^,^16^. In a word, the vacuole formation of the LV type involves in PSV coalescence, vacuole lumen acidification, and intracellular material mobilization^17^.

In the aleurone cells, the rupture of the large central vacuole results in the release of various hydrolytic enzymes into the starch endosperm; subsequently, the protoplast rapidly collapses and dies. The rupture of the vacuole and the subsequent release of various types of enzymes indicate that plant PCD has been initiated^18^. Furthermore, vacuolar collapse results in the loss of plasma membrane integrity and eventually leads to cell death^19^,^20^. It was suggested that the collapse of the large central vacuole is a crucial event in the PCD of plant cells, and vacuole-induced cell death is unique to plant cells^21^. In addition, the death of cereal aleurone cells is highly correlated with the extent of vacuolation, which means that the cell remains viable prior to vacuolation^22^. The phenomenon where one to three large vacuoles appear in aleurone PCD is known as vacuolation^23^,^24^. Vacuolation is an important event that reflects the process of cereal aleurone PCD^23^. In the late stage of PCD, the degradation of the cell nucleus is triggered by vacuole membrane rupture^25^. There is no doubt that tonoplast rupture and vacuole collapse are the important morphological features of PCD^26^.

The VPE is a cysteine protease with substrate specificity for the aspartic acid of YVAD^27^ and is localized in the plant vacuoles^2^.its optimum pH is acidic. Firstly, it was thought that the VPE was responsible for the processing and modification of proteins in vacuoles, i.e., the conversion of precursor proteins into mature forms, resulting in the formation of multifunctional proteins in vacuoles. Subsequently, it was found that the VPE could promote the hydrolysis of vacuole proteins and the rupture of the vacuole, playing a similar role to animal caspase in regulating PCD^28^,^29^. The VPE protein and its mRNA level increase at the beginning of the hypersensitive response (HR) reaction in the tobacco leaf, in which the cells showed typical PCD characteristics, and both the VPE inhibitor ESEN-CHO and the caspase-1 inhibitor Ac-YVAD-CHO inhibit the occurrence of PCD^30^. Therefore, VPE is regarded as a protease with caspase-1-like activity in plant^21^. CathB, a cysteine protease, displayed DEVDase (caspase-3-like) activity in *Arabidopsis thaliana*, but the CathB inhibitor CA-074 and the caspase-3 inhibitor DEVD-CHO strongly inhibited the DEVDase activity of AtCathB3^31^. In addition, AtCathB3 gene, encoding a cathepsin B-like protease, is highly induced during seed germination of *Arabidopsis thaliana*^32^. Furthermore, CathB with caspase-3-like activity plays a vital role in the PCD of plants^31^. Therefore, CathB is defined as a protease involved in cell death. Moreover, when the VPE genes were knocked-out, no characteristics pertaining to cell death were observed. After the leaf of wild-type tobacco exhibited a hypersensitive response (HR), the cells presented a series of typical features of PCD, such as cell shrinkage and vacuole membrane rupture. In leaves infected with Tobacco mosaic virus (TMV), VPE activation was initiated, leading to vacuole disruption and activation of PCD to prevent the proliferation of TMV^30^. Indeed, the proteases VPE and CathB with caspase-like activities exerted effects leading to the tonoplast rupture of plant PCD. In addition, proteases take part in the degradation of many cellular proteins, which determine the fate of cells^33^. The transformation from PSVs to LVs requires proteases to degrade and mobilize the storage proteins of PSVs^17^. Since LVs contain numerous hydrolytic enzymes used to degrade cytoplasmic components, only after large central vacuoles (typical LV) rupture, the hydrolytic enzymes are secreted from LVs, thus, the vacuole-induced cell death is correlated with the formation of LVs^34^,^35^. The implication is that the proteases play a vital role in the fusion of vacuoles in the PCD of plants.

Microfilaments (MFs), also called actin filament (AFs), are in a balance between polymerization and depolymerization, of which only the polymerization state plays a role in physiological functioning. Therefore, AF stabilizing and depolymerizing agents have usually been used to investigate their effects on the changes in cellular structure and various physical functions. AFs have been shown to be effectors as well as targets during PCD signaling^36^,^37^. The AF depolymerizing agent Latrunculin B (LB) induces DEVDase activity, thus LB plays an enhanced role in the PCD of *Papaver rhoease* pollen^38^. However, the AF stabilizing agent phalloidin prevents nuclear fragmentation in porcine kidney proximal tubule cells, implying that the cell death is inhibited^39^. Mounting evidence shows that AFs are associated with the fusion and dynamic changes of vacuoles. After tobacco protoplasts were treated with the AF depolymerizing agent cytochalasin B (CB), the dynamic wave structure on the surface of vacuoles disappeared; by contrast, the dynamic structure was not changed after treatment with the microtubule depolymerizing agent Oryzalin^40^. All of the above results indicate that the dynamic structure of vacuoles is regulated by AFs^41^. In addition, a tubular vacuole was formed during tobacco BY-GV 7 mitosis, whereas the AF depolymerizing agents bistheonellide A (BA) or CB led to the disappearance of the tubular vacuole. This indicated that AFs are involved in maintaining the state of tubular vacuoles in tobacco cell mitosis^42^. The AF depolymerizing agent CD also inhibited the dynamic change of the barrel and lamellar structure of vacuoles in transgenic *Arabidopsis*^43^. More importantly, AF depolymerization contributed to vacuole fusion in yeast^44^.

In this study, we investigated morphological changes in vacuoles and the effect of vacuolation on PCD. Sequential observations in aleurone cells revealed that the rupture of the large central vacuole resulted in the disruption of the plasma membrane, followed by the disintegration of the nucleus and PCD. The depolymerization in AFs influenced the degree of vacuolation and the process of PCD. Furthermore, it is hypothesized that the VPE accelerates PCD due to the induction of vacuole coalescence and the rupture of vacuoles. These results contribute knowledge to fields such as biology and plant morphological anatomy, as well as provide the theoretical basis for increasing the quality of rice seed germination.

## Results

### Two types of vacuole fusion occurred in rice aleurone layers

The viability and death of aleurone cells were monitored by simultaneously staining living and dead cells with the fluorescent dyes FDA and FM4-64. FDA is taken up by living cells and emits green fluorescence. FM4-64 penetrates slowly into live cells, but accumulates rapidly in dead cells and emits orange/red fluorescence^45^,^46^,^47^,^48^,^49^.

Before the large central vacuole ruptured and collapsed, the cell emitted FDA green fluorescence, indicating it was live. However, when the rupture and collapse of the vacuole occurred, the permeability of the plasma membrane increased, after which the protoplast began to shrink, and FM4-64 red fluorescence observed within the cell showed that cell death had been triggered (see Supplementary Fig. S1). Therefore, the large central vacuole is a critical morphological characteristic and significant marker prior to the occurrence of PCD triggered by vacuoles rupture in rice aleurone layers.

**Figure 1:**
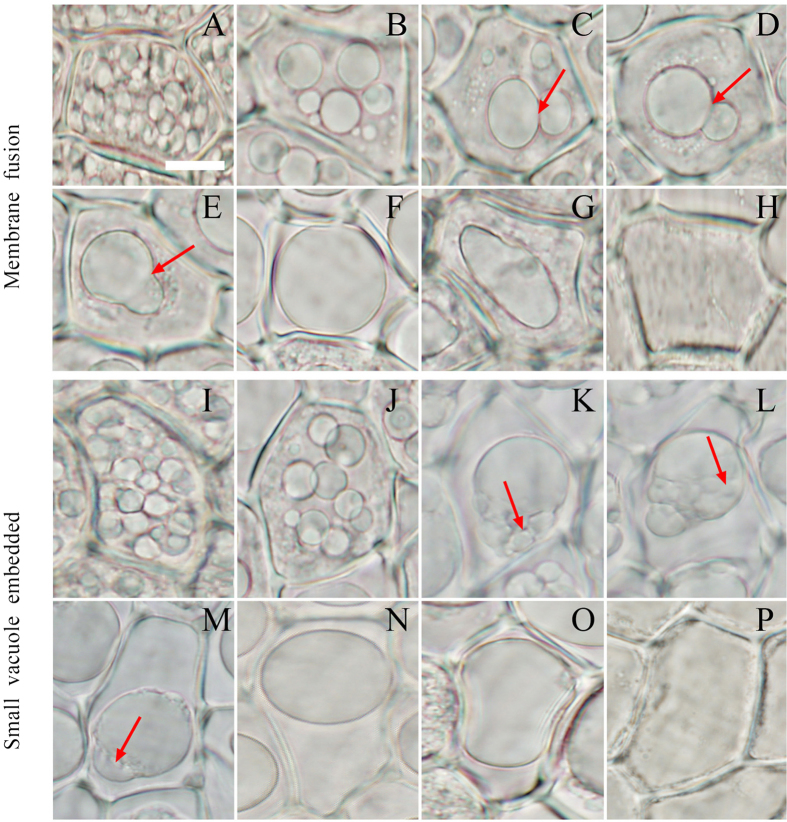
The integration and transformation of vacuoles in the PCD of aleurone layers. The aleurone layers stripped from grains cultured for different times were observed using fluorescent microscopy, and the vacuoles exhibited two types of fusion. (**A–H**) Vacuole fusion through membrane fusion; arrows indicate the mutual contact section (**C,D**) and the mutual fusion section (**E**) of the tonoplast. (**I–P**) Vacuole fusion through embedded fusion; arrows indicate the small vacuole starting to enter the larger vacuole (**K**), the small vacuole in the larger vacuole (**L**), and the trace of the small vacuole in the larger vacuole (**M**). The assay was performed at least three times and a representative aleurone layer section is shown. The bar represents 10 μm.

The small PSVs distributed in the aleurone cells at the early imbibition of rice seeds (Fig. 1A,I). However, the formation of larger vacuoles through the coalescence of small vacuoles was observed in the aleurone cells, which may have resulted from two types of fusion of vacuoles in the aleurone cells (Fig. 1). The first type is that a large vacuole and a smaller vacuole co-existed in a cell, which touched each other through the membrane (Fig. 1C,D, arrow), after which they fused together (Fig. 1E, arrow). The larger vacuole became larger, and the coalescence of the two vacuoles gradually resulted in the formation of a large central vacuole (Fig. 1F). Secondly, the large central vacuole underwent the changes of deformation and elongation (Fig. 1G and see Supplementary Fig. S1) before it ruptured (see Supplementary Fig. S1) and disappeared (Fig. 1H and see Supplementary Fig. S1). Ultimately, the protoplast dissolved and the cell died^48^; this type of fusion is referred to as membrane fusion. In the other type of fusion, a large vacuole (obviously LV) and a few small vacuoles appear together in a cell (Fig. 1K, arrow). The small vacuoles are clearly embedded in the larger vacuole, or the larger vacuole swallows the small vacuoles, after which the small vacuoles are clearly observed within the larger vacuole (Fig. 1L, arrow). Further, these small vacuoles are gradually digested and dissolved in the lumen of the larger vacuole (Fig. 1M, arrow), finally forming a large central vacuole (Fig. 1N,O). Similarly, the vacuole ruptures and disappears (Fig. 1P), resulting in cell death. This is referred to as embedded fusion. In addition, the loss of the boundary between the vacuole and the cytoplasm occurred following the disappearance of the large central vacuole (Fig. 1H,P), which is thought to be a marker of tonoplast rupture^25^. In brief, these two types of vacuole fusion appeared in the aleurone cells before a large central vacuole formed.

### The cell death of aleurone layers started in the section close to the embryo and extended to the section far from the embryo

Acridine orange (AO) penetrates all cells, living and dead. Nuclei in living cells appear green under fluorescence when AO is bound to DNA^50^,^51^, and also AO stains vacuoles emitting green fluorescence^52^.

**Figure 2:**
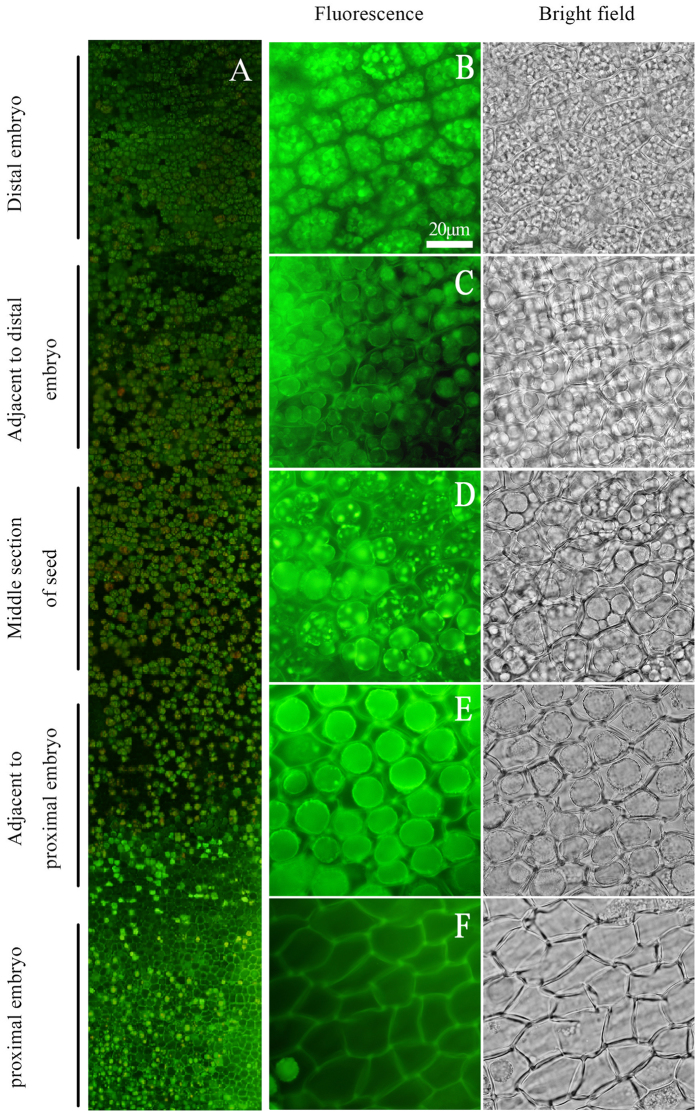
Proximal-to-distal gradient of programmed cell death in the aleurone layers of rice seeds. The aleurone layers were longitudinally stripped from the rice grains cultured for 5 d, and changes in cells after staining with 8.5 μg mL^−1^ AO were examined using fluorescence microscopy. Live cells appear green due to AO fluorescence. (**A**) The intensity changes in AO green fluorescence in the aleurone layer. (**B–F**) Corresponded to the intensity changes of AO green in the aleurone layers of the distal embryo, adjacent to the distal embryo, middle section of the seed, adjacent to the proximal embryo and the proximal embryo, respectively; the bar represents 20 μm. The assay was performed at least three times and a representative aleurone layer section is shown.

The aleurone layers were longitudinally stripped from the rice seeds cultured for 5 d. After these layers were stained with AO, the development of vacuolation differed in the section near the embryo and in the section far from the embryo, observed using a fluorescent microscope (Fig. 2A). The aleurone cells were filled with small vacuoles in the distal embryo section, and their contents had a strong fluorescence intensity (Fig. 2B). By contrast, the vacuoles of the cells in the section adjacent to the distal embryo began to fuse into larger vacuoles, but the number of formed vacuoles was reduced, and these cells contained fewer contents than those cells in the distal embryo section (Fig. 2C). In addition, the former emitted smaller fluorescence spots than the latter (Fig. 2B,C). Two or three larger vacuoles occurred within a cell in the middle section of the seed under bright field, indicating considerable vacuolation, and their fluorescence spots were much bigger (Fig. 2D). It indicated that there was a positive correlation between the size of fluorescence spot and the degree of vacuolation in the aleurone cells. The aleurone cells in the section adjacent to the proximal embryo exhibited large central vacuoles, and only the large central vacuoles emitted fluorescence (Fig. 2E). The presence of lumens but no green fluorescence spots indicated that the vacuoles of the aleurone cells in the proximal embryo section disappeared (Fig. 2F). In summary, the vacuolation of the aleurone cells occurred from the proximal embryo section to the distal embryo section (Fig. 2A); therefore, the cell death of the aleurone layers also started in the proximal embryo section and extended to the distal embryo section.

### The cell death of aleurone layers started from the inner layer and extended to the outer layer in the transverse section of the rice seeds

**Figure 3:**
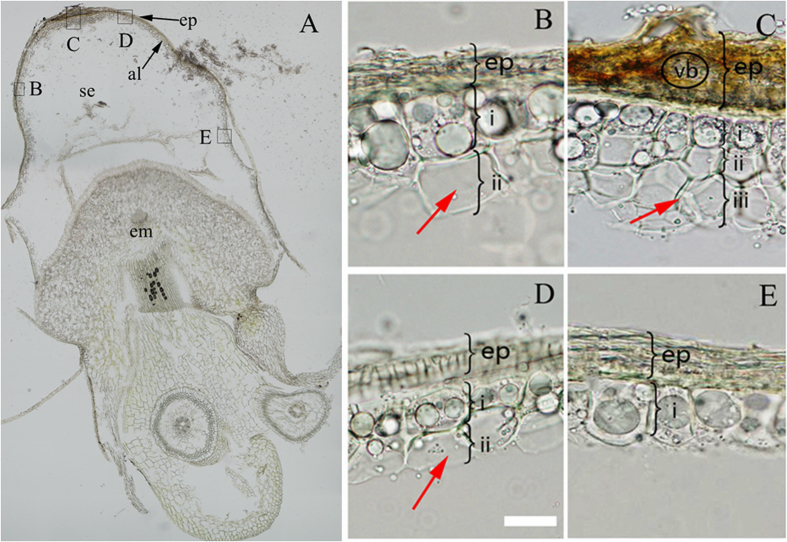
Inner-to-outer gradient of programmed cell death in the aleurone layers of rice seeds. (**A**) The transverse section was laterally cut rice grains cultured for 5 d (with embryo); (**B–E**) Details corresponding to A. Analyses were repeated at least three times on independent biological samples, and representative results are shown. The bar represents 20 μm. al, aleurone layer; em, embryo; se, starch endosperm; ep, episperm + pericarp; vb, vascular bundle. In panel B and D: i, the outermost layer; ii, the innermost layer; and in panel C: i, the outermost layer; ii, the middle layer; iii, the innermost layer.

Most cereal seeds only contain one layer of aleurone cells enveloping the starch endosperm; however, the number of layers in the rice seed varied in the different sections. In the transverse section cut across the embryo of the rice seeds (Fig. 3A), a large and obvious vascular bundle appeared at the back of the rice seed, where three layers of aleurone cells were observed (Fig. 3C). It is speculated that the transportation of additional material for grouting results in the structure of this section, whereas in both sides near the vascular bundle, the aleurone cells gradually transformed into two layers (Fig. 3D). The alternation between a single layer of cells and two layers of cells was observed in a position far from the vascular bundle (Fig. 3B). In addition, the aleurone layers adjacent to embryo were mostly one layer of cells (Fig. 3E). However, the cell morphology was different in the different sections during germination for 5 d. Firstly, the large central vacuole only occurred in one layer of aleurone cells (Fig. 3E); moreover, in the zones of aleurone with two layers, the cells adjacent to the starch endosperm were completely degraded, the lumen was dead (Fig. 3B,D, arrows), and larger vacuoles or large central vacuoles occurred in the cells adjacent to the episperm. The aleurone cells of the inner-most layer at the back of the seed had lumens surrounded by cell wall (Fig. 3C, ii and iii, arrow), indicating these cells were dead, whereas the cells of the outer-most layer contained large central vacuoles and unquestionably, the cells were still live (Fig. 3C,i). Therefore, the death of aleurone cells started from the inner layer (i.e., adjacent to the starch endosperm) and then gradually transitioned to the outer layer (i.e., adjacent to the episperm); specifically, the PCD of the aleurone layers occurred from the inner layer to the outer layer.

### The rupture of the vacuole induced an increase in the plasma membrane permeability in the PCD of the aleurone layers

Evans blue (EB) cannot pass through the plasma membrane of living cells but can leak into damaged membranes and enter into cells^53^,^54^. EB blue images were examined for cell death under a light microscope^55^, and EB red fluorescence was observed for cell death under a fluorescence microscope^56^. Therefore, live and dead cells are determined by observing the integrity of the plasma membrane.

**Figure 4:**
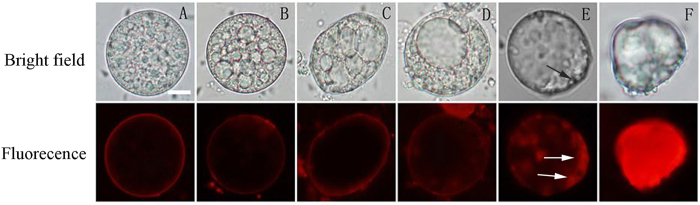
The detection of protoplast membrane permeability. The images of protoplasts stained with 10 μg mL^−1^ Evans blue (EB) were observed at different developmental stages under fluorescence microscopy. (**A–D**) EB red fluorescence remained on the plasma membrane (PM), and the corresponding bright images showed the intact vacuole. (**E,F**) The vacuole disappeared under bright field. EB red fluorescence penetrated the PM (E, white arrows), and the corresponding bright image showed the vague boundary between the cytoplasm and the PM (E, black arrow). A mass of EB red fluorescence appeared (**F**), and the protoplast shrunk in the corresponding bright image. Both experiments were repeated, and similar images were obtained. The bar represents 10 μm.

Before the large central vacuoles disappeared, the red fluorescence emitted from EB only remained on the surface of the plasma membrane and did not pass through the membrane, which indicated that the protoplast was alive and the membrane had selective permeability, thus the membrane was intact (Fig. 4A–D). However, after the large central vacuole disappeared, the boundary between the cytoplasm and the plasma membrane was no longer obvious under bright field (Fig. 4E, black arrow). In addition, red fluorescence was emitted from the inside of the protoplast membrane, i.e., EB had passed through the plasma membrane into the protoplast (Fig. 4E, white arrow). These results showed that the plasma membrane lost its integrity, resulting in the start of protoplast death. Then, the protoplast shrunk into a mass (Fig. 4F). Finally, the shrunken protoplast was filled with red fluorescence EB (Fig. 4F), which indicated that the protoplast had died. The above results confirmed that the rupture of the vacuole resulted in the loss of plasma membrane integrity, which in turn resulted in the death of the protoplast.

### The rupture of the vacuole occurred prior to the degradation of the nucleus in the PCD of the rice aleurone layers

4′, 6-diamidino-2-phenylindole (DAPI) can pass through the intact plasma membrane and emit strong blue fluorescence by combination with most A and T bases of DNA^57^. Therefore, it is often used to dye ordinary nuclei and specific double-stranded DNA and can be used to investigate morphological changes of nuclei under a fluorescent microscope.

**Figure 5:**
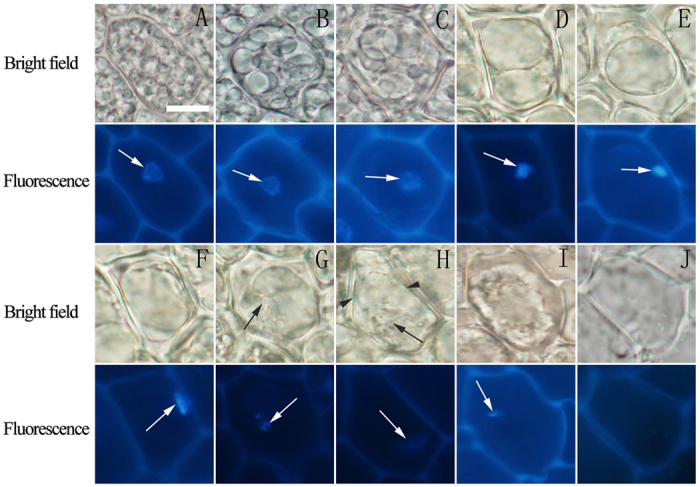
Nuclear morphological changes of aleurone cells at different stages of cell death. The morphology of nuclei in the aleurone cells stained with 1 μg mL^−1^ DAPI was observed by fluorescence microscopy. (**A–J**) The nuclei stained with blue fluorescence DAPI (arrows) and the corresponding images under bright field. Both experiments were repeated, and similar images were obtained. The bar represents 10 μm.

Aleurone cells filled with small PSVs with a granular structure at the early stage of germination, while the nucleus located in the center of the cell was large and round, emitting a uniform and bright blue fluorescence (Fig. 5A, white arrow). Subsequently, larger vacuoles occurred within the cell, and the nucleus remained near the center of the cell (Fig. 5B,C, white arrows). When only two large vacuoles were present in the cell, the nucleus located between the two vacuoles still emitted uniform blue fluorescence (Fig. 5D, white arrow). A large central vacuole appeared in the aleurone cell, which occupied most of the cell volume, with the result that the nucleus was pushed to the edge of the cell, and its volume was small and oval, emitting strong blue fluorescence (Fig. 5E, white arrow). With the large central vacuole elongated and deformed, the shape of the nucleus changed, presenting an elongated rod (Fig. 5F, white arrow). After the rupture of the large central vacuole occurred (Fig. 5G, black arrow), the nucleus became smaller and incomplete, emitting less blue fluorescence (Fig. 5G, red arrow). When the boundary between the cytoplasm (Fig. 5H, black arrow) and vacuole disappeared and the plasma membrane separated from the cell wall (Fig. 5H, black arrowheads), the nucleus was diffused with a weak blue fluorescence (Fig. 5H, red arrow). Then, the integrity of the plasma membrane was lost, the protoplast shrunk (Fig. 5I), but the nucleus was not degraded and still emitted a very weak blue fluorescence (Fig. 5I, red arrow), suggesting that only a small amount of DNA remained. Accompanied by the disappearance of the protoplast in the cell, the cell became a lumen, i.e., a dead cell with a wall, because the nucleus had been degraded and disappeared at that time, without any fluorescence been emitted from the cell (Fig. 5J). The above results confirmed that the rupture and disappearance of the large central vacuole occurred prior to the degradation of the nucleus.

### AF-depolymerizing agent and AF-stabilizing agent induced or inhibited vacuolation in rice aleurone layers

**Figure 6:**
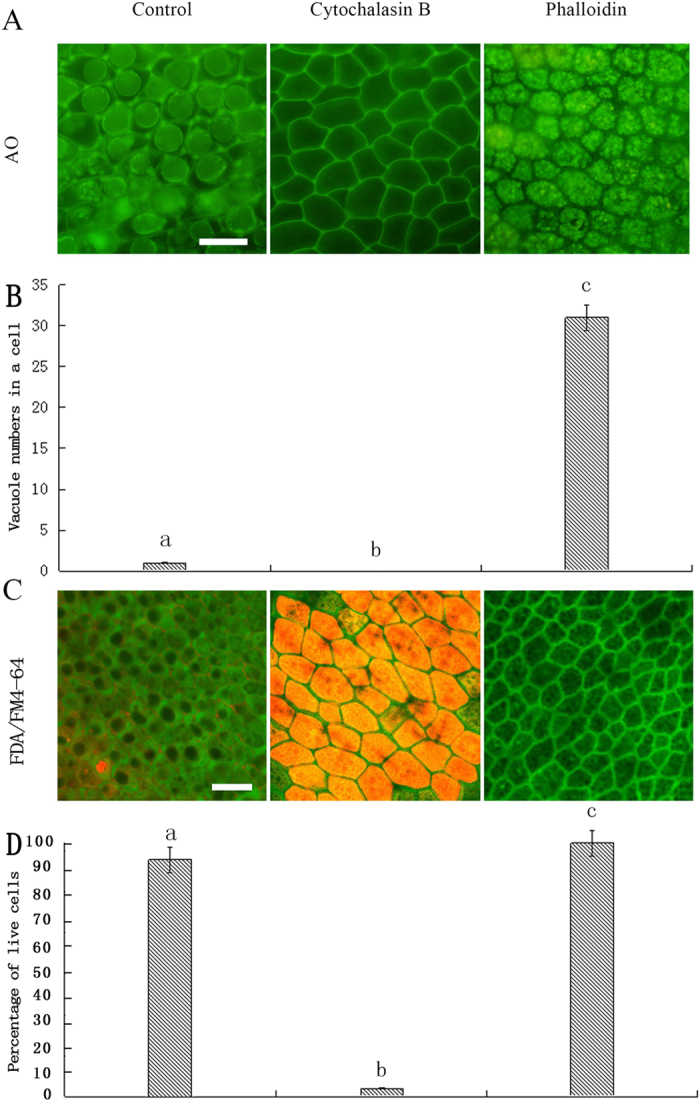
Antagonistic effects of CB and phalloidin on vacuole coalescence. Rice aleurone layers were isolated from seeds imbibed for 2 d and then incubated with distilled water, 10 μg mL^−1^ CB and 10 μg/ml phalloidin for 5 d. Finally, the layers were examined for vacuolation after 8.5 μg mL^−1^ AO or 2 μg L^−1^ FDA/1 μg L^−1^ FM4-64 staining using LSCM. (**A**) Live cells exhibit AO green fluorescence. (**B**) Statistical analyses were conducted on the vacuole number per cell. (**C**) Green fluorescence (FDA) stains the live cells and orange fluorescence (FM4-64) stains the dead cells. (**D**) Viability of the cells was quantified from at least four aleurone layers. The assay was performed at least three times and a representative aleurone layer section is shown. Data represent the mean ± SD from three independent biological replicates, and different letters indicate a significant different at p < 0.05 according to Duncan’s multiple range test. The bar represents 50 μm.

To verify the effect of the dynamic change in AFs on the aleurone layer PCD, the aleurone layers were treated with distilled water, AF depolymerizing agent cytochalasin B (CB) or stabilizing agent phalloidin for 5 d. The results showed that only a large central vacuole appeared in the cells of the rice aleurone layers treated with distilled water by using the fluorescence reagent AO and LCSM (Fig. 6A), whereas the protoplast of aleurone cells treated with CB had disappeared (Fig. 6A). The vacuoles of the aleurone cells treated with phalloidin were still small PSVs (Fig. 6A), and these PSVs filled the whole cell. The statistical results showed that each cell contained more than 30 vacuoles in the phalloidin treatment (Fig. 6B).

We then determined the viability of aleurone cells treated with distilled water, CB or phalloidin by using double fluorescence reagent FDA and FM4-64 combined with LSCM (Fig. 6C). After treatment for 5 d, 96.07% of aleurone cells treated with distilled water emitted FDA green fluorescence; however, the central region of cells occupied by large central vacuoles did not emit green fluorescence. Nevertheless, these cells were still alive. However, 91.67% of the aleurone cells treated with CB emitted FM4-64 orange fluorescence, which indicated that the cells were dead (Fig. 6C,D). In contrast, those cells treated with phalloidin emitted dense FDA green fluoresce and accommodated numerous small vacuoles, which clearly indicated that the cells were alive (Fig. 6C). We proved that the depolymerized AFs accelerated the process of PCD; by contrast, the stabilized AFs inhibited the fusion of vacuoles and delayed PCD.

### The inhibitors of VPE and CathB retarded the fusion of vacuoles in the rice aleurone layers

**Figure 7:**
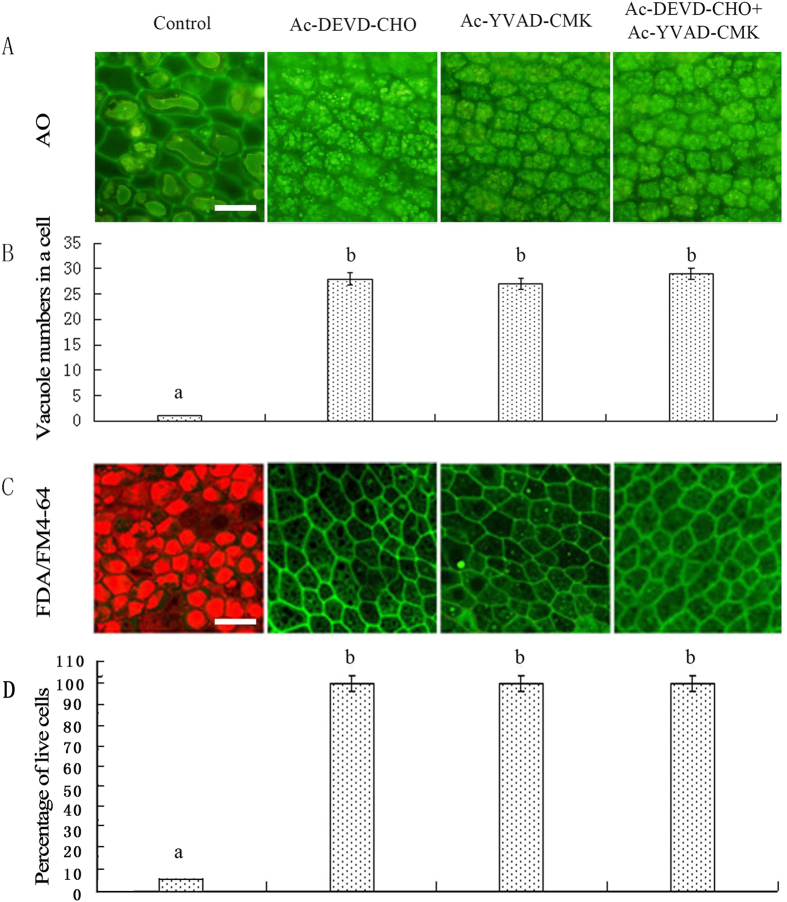
Ac-YVAD-CMK and Ac-DEVD-CHO slow the process of vacuolar coalescence. Rice aleurone layers were isolated from seeds imbibed for 2 d and then incubated with distilled water, 100 μM Ac-DEVD-CHO, 100 μM Ac-YVAD-CMK or 100 μM Ac-DEVD-CHO plus 100 μM Ac-YVAD-CMK for 7 d. Finally, the layers were examined for vacuolation after 8.5 μg mL^−1^ AO or 2 μg L^−1^ FDA/1 μg L^−1^ FM4-64 staining using LSCM. (**A**) Live cells exhibit AO green fluorescence. (**B**) Statistical analyses were conducted on the vacuole number per cell. (**C**) The live cells exhibit FDA green fluorescence and the dead cells exhibit FM4-64 red fluorescence. (**D**) Viability of the cells was quantified from at least four aleurone layers. The assay was performed at least three times, and a representative aleurone layer section is shown. Data represent the mean ± SD from three independent biological replicates, and different letters indicate a significant different at p < 0.05 according to Duncan’s multiple range test. The bar represents 50 μm.

After the aleurone layers were treated with distilled water, the VPE inhibitor Ac-YVAD-CMK or the CathB inhibitor Ac-DEVD-CHO for 7 d, the large central vacuoles of aleurone cells in the distilled water treatment were elongated and deformed, and some cells appeared to be lumens, which showed these cells were dead (Fig. 7A). In contrast, the aleurone cells emitted dense FDA green fluorescence in the treatments with Ac-DEVD-CHO or Ac-YVAD-CMK alone or together, and the vacuoles contained numerous small PSVs; therefore, the morphology of these vacuoles indicated that they were far from vacuolation, and each cell contained more than 30 vacuoles (Fig. 7B). These results indicated that the VPE inhibitor and CathB inhibitor inhibited the fusion of vacuoles in the aleurone layers.

Furthermore, the cell viability in the different treatments was detected using double fluorescence reagents FDA and FM4-64 combined with LSCM. A total of 94.98% of the aleurone cells treated with distilled water exhibited strong FM4-64 red fluorescence, which showed that the cells had died (Fig. 7C,D). All of the aleurone cells treated with the VPE inhibitor and CathB inhibitor exhibited FDA green fluorescence, respectively, indicating the cells were alive (Fig. 7C,D). The above results indicated that the VPE inhibitor and CathB inhibitor suppressed the fusion of vacuoles and delayed the PCD of the rice aleurone cells.

## Discussion

There are no macrophages in plants; the unwanted material for PCD is only degraded through vacuole-released hydrolytic enzymes. Therefore, vacuoles play an important role in plant PCD. During the germination of cereal seeds, the embryo-secreted GA induces the PCD of aleurone cells, which results in the death of the aleurone cells and in turn, releases a series of hydrolases to degrade storage substances in starch endosperm, further providing nutrition for seed germination^58^. The initial aleurone cells were filled with many small PSVs; these PSVs fused together to form larger vacuoles. In this fusion process, the volume of the vacuoles increased, but the number of vacuoles was reduced. Subsequently, vacuolation appeared, and a large central vacuole formed. Then, the large central vacuole orderly underwent extension, rupture, and disappearance, with the protoplast collapsing into a mass. Ultimately, the cell was completely degraded and the remaining lumen was only surrounded by a cell wall^48^ (see Supplementary Fig. S1). Fath *et al*.^58^ reported similar results for barley aleurone layers. Before the large central vacuole elongated and deformed, the cell was alive. When the large central vacuoles ruptured and disappeared, the lumen of the cell filled with FM4-64 orange/red fluorescence, indicating that the cell lost its viability (see Supplementary Fig. S1). Finally, the shrunken protoplast was completely degraded; the cell lumen did not contain any contents and did not emit fluorescence (see Supplementary Fig. S1), which meant that the cells were dead. Bethke *et al*.^22^ showed that vacuoles play an important role in the PCD of barley aleurone, and only cells with high vacuolation resulted in PCD. Therefore, the degree of vacuolation can reflect the process of PCD.

Most vacuoles were elongated and deformed in the aleurone cells of rice seeds cultured with distilled water for 7 d; further, 24.48% of the cells died. By contrast, almost all vacuoles were small PSVs in the cells treated with the vacuole fusion inhibitor E-64d, and they were living cells (see Supplementary Fig. S2). This indicated that vacuole fusion was blocked, and vacuolation did not occur. The extended development of vacuolation will delay the occurrence of PCD in the rice aleurone layer. The results showed that E-64d significantly inhibited the vacuolation of the aleurone cells. Vacuole fusion is a critical event through which a large central vacuole forms within a cell^10^.

Mansfield and Briarty^59^ also showed that numerous PSVs fused to form an LV in the germination and seedling development of *Arabidopsis* after most protein reserves were mobilized. Smaller vacuoles merge into larger vacuoles or large central vacuoles through two types of fusion, i.e., membrane fusion and embedded fusion. Through these two types, vacuoles gradually merge into a large central vacuole, and membrane fusion may be the main fusion type wherein small PSVs merge into larger PSVs (Fig. 1B,J). By contrast, embedded fusion represents the fusion between smaller and larger vacuoles only during the later stage of cells (Fig. 1K–M). The two types of fusion result in gradual transformation into a large central vacuole of the LV type before cell death is triggered. Therefore, the two types of vacuole fusion can also be regarded as the two methods of transforming PSVs to LVs. A large central vacuole is a typical morphological feature that can be easily identified in the vacuole-induced PCD of cereal aleurone layers. Vacuole fusion is an essential process for vacuolation. Cao *et al*.^60^ suggested that cells containing more than three vacuoles with diameters >2 μm were defined as vacuolated cells by means of quantification. In our experiments, we identified a large central vacuole as a morphological indication of vacuolation, which should be more typical than multiple larger vacuoles. Therefore, this indicator of vacuolar morphology may provide us with convenient to investigating the mechanism of PCD in rice aleurone cells.

The vacuolation degree of aleurone cells was different in the different sections of the rice seeds for 5 d of culture. The vacuoles of the aleurone cells adjacent to the embryo fully disappeared (Fig. 2F), whereas, the aleurone cells near the middle of the embryo contained large central vacuoles, i.e., in a high state of vacuolation (Fig. 2E). Interestingly, the cells in the distal embryo contained a large number of small PSVs, which indicated that these cells had not undergone vacuolation (Fig. 2B). These results also confirmed that the vacuolation and PCD of aleurone cells started near the embryo and then gradually developed to the distal embryo. Wang *et al*.^61^ and Domínguez *et al*.^62^ also suggested that the PCD of cereal aleurone layers initially occurred near the embryo. The process of aleurone PCD is controlled by the location of embryo, which may be due to the embryo producing GA^22^,^46^. Furthermore, the aleurone PCD gradually occurred from the inner to the outer in the transverse section of seed, which indicated that the occurrence of PCD exhibits a certain position effect. The cells of the inner-most aleurone layer (i.e., adjacent to the starch endosperm) died initially, and the cell death of the outer-most layer (i.e., adjacent to the episperm) occurred last (Fig. 3). Based on the above results, the cell death of the rice aleurone layers exhibited a characteristic spatio-temporal mode.

It is accepted that the vacuolar collapse is a crucial event in the cell death of plants^21^. The rupture of the vacuole results in the rapid acidification of the intracellular space and the hydrolysis of residual cell contents^63^. The integrity of the plasma membrane is maintained until the rupture of the vacuole^35^. In this study, when a large central vacuole appeared in the cytoplasm, the protoplast was still alive; however, the plasma membrane permeability increased with the disappearance of the large central vacuole. The result shows that cell death is executed immediately after the initiation of the rupture of the large central vacuole. Thereafter, the plasma membrane lost its boundary with the cytoplasm (i.e., the plasma membrane ruptured) and shrank. Finally, the protoplast shrank into a mass, i.e., the protoplast was dead (Fig. 4), which demonstrated that vacuolar rupture occurred prior to the loss of plasma membrane integrity in the PCD of the aleurone cells. When the large central vacuole elongated and deformed, the shape of the nucleus changed (Fig. 5F). Finally, after the vacuole ruptured, and the nucleus became smaller; and then slowly disappeared (Fig. 5G–J). Obara *et al*.^25^ confirmed that tonoplast rupture resulted in the degradation of the nucleus in the mesophyll cells during differentiation of tracheary elements in *Zinnia*. A new type of nucleic enzyme, ZEN1, was released from the ruptured vacuoles, which then degraded DNA, resulting in cell death^64^. The contents of cells are degraded during plant PCD, and it is speculated that PCD may be related to hydrolases released by the ruptured vacuole^65^,^66^. Because the ruptured vacuole releases various hydrolytic enzymes, vacuole rupture is used as an indicator of PCD initiation^18^. In this study, it was also observed that when the plasma membrane began to shrink inward and a mass of shrunken protoplast separated from the wall of the cell, the small irregular nucleus was still within the cell (Fig. 5I, arrow), which showed that the nucleus had not yet collapsed and that the vacuoles and plasma membrane had degraded before the nucleus dismantled. Similarly, high vacuolation and the loss of plasma membrane integrity occurred in wheat aleurone cells, although the nucleus was still evident^62^. Vacuolation is the first phenomenon in the PCD of barley aleurone cells, followed by the loss of integrity of the plasma membrane, DNA degradation and finally cell death^58^. Therefore, the degradation and dissolution of the cell contents, including the cytoplasm and nucleus, in the PCD of aleurone cells are triggered by the rupture of the vacuole.

AFs are involved in the regulation of vacuolar dynamics^67^. The AF stabilizing agent may inhibit the vacuole fusion of cells, resulting in the inhibition of stomatal opening^41^. In addition, the AF stabilizing agents latrunculin B and jaspla-kinolide inhibited the vacuole fusion of yeast^68^. In this study, the AF depolymerizing agent CB obviously promoted the disappearance of vacuoles, and then accelerated cell death. On the contrary, coalescence and enlargement of PSVs occurred more slowly in the layers treated with the stabilizing agent phalloidin; accordingly, the stabilizing agent delayed vacuolation and PCD (Fig. 6). Therefore, aleurone cells are likely to regulate the changes in vacuole morphology by changing the dynamic structure of the AFs, which affect the process of PCD. All of the above results confirm that AFs are required for the formation of the large central vacuole.

Studies have shown that the progression of PCD may require the activation of caspase-like activities in plants^69^,^70^,^71^. Importantly, the inhibitors of proteases with caspase-like activities play a key role in preventing the coalescence of small vacuoles in the rice aleurone cells. In this study, both the VPE specific inhibitor Ac-YVAD-CMK and the CathB inhibitor Ac-DEVD-CHO significantly inhibited the mutual fusion of vacuoles and decreased the degree of vacuolation in the aleurone cells, which delayed PCD (Fig. 7). Apparently the proteases are required to participate in the transformation from PSVs to LVs^17^,^72^. Intriguingly, E-64d, an inhibitor of vacuole fusion^73^, also a cystein cathepsin inhibitor^74^, prevents the proteolytic activity of CathB^74^. However, E-64d does not inhibit the activities of VPE and caspase-1^27^. In our study, E-64d delayed the occurrence of the large central vacuoles and the process of PCD (see Supplementary Fig. S2). However, when the activities of VPE and CathB were not inhibited such as in the control in Fig. 7, the small vacuoles could merge into the large central vacuoles, resulting in a sequence of events that included vacuole rupture, cytoplasm degradation, and nucleus collapse, followed by cell death.

Hatsugai *et al*.^19^ and Nakaune *et al*.^75^ suggested that the disruption of the vacuole may be mediated by the VPE. Activated VPE may process protein kinase in the vacuole, promoting the rupture of vacuole, which triggers cell PCD. Then, the VPE continually processes the enzymes involved in cell PCD and degrades cell material, which leads to the occurrence of plant PCD^76^. Therefore, the VPE is able to initiate PCD and induce the hydrolysis enzyme for the degradation of the cell contents. By contrast, the aleurone layers treated with VPE inhibitors do not undergo cell death. These observations suggest that VPE and CathB may promote coalescence, accelerating the process of vacuolation, and that VPE is a key molecule in triggering vacuolar collapse in the PCD^2^,^77^.

In summary, we confirmed the sequential changes of vacuole rupture, plasma membrane integrity loss, nucleus collapse and cell death in the rice aleurone cells, and also confirmed the fusion of vacuoles needed by the formation of the large central vacuole. The rupture of the large central vacuole initiated the PCD of the aleurone layers. However, further research is required to explain the regulation mechanism of the VPE and CathB in the fusion of the vacuole.

## Materials and Methods

### Plant materials and chemicals

Grains of rice (*Oryza sativa* L.) were sterilized in 0.1% (v/v) potassium permanganate for 5 min and washed three times with sterile water. These sterile grains were cultured in a Petri dish containing two layers of filter paper soaked with sterile water at 25 °C for 2 d, and were then transferred to a 27 °C/25 °C growth chamber with 16-h light photo-period. The grains were cultured for different times according to the experimental requirement.

All chemicals were purchased from Sigma (St Louis, MO, USA), unless stated otherwise.

### Determination of cell viability and vacuole numbers per cell

The aleurone layers at different culture times used to detect the viability of the cell were prepared and detected as described previously^45^. The layers were stained with fluorescein FDA (2 μg∙mL^−1^ in 20 mM CaCl_2_) for 15 min, followed by 20 mM CaCl_2_ to remove background fluorescence, stained with FM4-64 (1 μg∙mL^−1^ in 20 mM CaCl_2_) for 3 min, then washed with 20 mM CaCl_2_. Images of the layers were captured with a laser scanning confocal microscope (LSCM, FV1000, Olympus), and at least three different aleurone layers were measured per treatment. The percentage of viable cells was determined by counting the number of live and dead cells in different fields, and the numbers were averaged for each half-seed.

In addition, the aleurone layers in the central part of the seeds were stripped, and then changes in the vacuoles of the aleurone cells were observed using laser scanning confocal microscopy (LSCM). Statistical analyses were conducted on the vacuole numbers of a single cell.

### Preparation of aleurone layers for pharmacology

The aleurone layers were separated from the central parts of rice grains immersed in distilled water for 2 d; they, in turn, were incubated with distilled water, 100 μM Ac-DEVD-CHO, or 100 μM Ac-YVAD-CMK for 7 d, and/or incubated in distilled water, 10 μg mL^−1^ Phalloidin, or 10 μg mL^−1^ cytochalasin B (CB) for 5 d, after which these treatments were stained with 8.5 μg mL^−1^ AO. The cell morphology of the layers was observed using a fluorescence microscope, and then the live and dead cells were examined.

### Observation of frozen sections

The rice seeds stripped from grains cultured in distilled water for 5 d were placed on a fast-freezing table and frozen for 14 h. The frozen seeds were placed on the Peltier element and were then embedded in glue for approximately 20 min. Then, the embedded blocks were clamped on the holder on the frozen section machine, and then the slices were cut (approximately 12 μm) from the blocks. Finally, the structure and morphology of the aleurone cells were observed with fluorescence microscopy and photographed (Olympus BX51, digital imaging system Olympus DP71).

### Morphological detection of aleurone cells in intact rice grains

The aleurone layers were longitudinally stripped from the rice grains after they were cultured for 5 d, and then the layers were stained in an 8.5 μg mL^−1^ AO solution for 15 min. The morphology of aleurone cells in the different sections was investigated using a fluorescence microscope (Olympus BX51, digital imaging system Olympus DP71) with an excitation wavelength of 488 nm and an emission of 515 nm, after which the images were photographed.

### Detection of nucleus fluorescence

The aleurone layers were stripped from the rice grains at the different stages of germination, and then these layers were shocked for 20 min to remove residual starch particles on the surface of their cells. The layers were placed in 1 μg mL^−1^ DAPI, stained for 1 h, and were then washed with phosphate buffer solution 4–5 times, for 5 min each time. Then, images of the nucleus morphology of the layers at different stages were obtained using a fluorescent microscope (Olympus BX51, digital imaging system Olympus DP71) under an excitation filter wavelength of 365 nm and a blocking filter wavelength of 420 nm.

### Detection of plasma membrane integrity

The embryos and tails of grains cultured for 4 d were removed and soaked in 0.25% NaClO solution for 40 min, washed several times with sterile water, placed in 0.01 mM HCl for 10 min to remove residual NaClO, and were then washed several times again using sterile water. The treated half-seeds were cut along the ventral surface, the aleurone layers were isolated and then collected in a medium. Subsequently, these layers were transferred to improved B5 medium containing 5% cellulase and 1% macerozyme, and then these layers were shocked for at least 1 h at room temperature to remove the excess starch grains and ensure the enzyme solution was fully dissolved the aleurone layers. The shocked layers were transferred to improved B5 culture solution containing 5% celluase, 1% macerozyme, and 1 mM DTT (containing 150 U mL^−1^ nystain and 0.1 mg mL^−1^ Cefotaxime) for 48 h under static culture, which was replaced with a improved B solution during this period. The aleurone protoplasts were scraped from the layers with a surgical blade; these layers were centrifuged for 3 min at 500 rpm, and then the protoplasts at the bottom were collected and re-suspended in modified B5 medium, after which impurities were removed from these layers using a sieve with a 100-μm pore size. The protoplasts were collected and cultured in modified B5 medium for 3 d. Then, the cultured protoplasts were stained in 10 μg mL^−1^ Evans solution for 5 min and cleaned using modified B5 medium several times. The protoplasts cultured at different periods were chosen for observation, and images of plasma membrane integrity were obtained using a fluorescence microscope (Olympus B × 51, digital imaging system: Olympus DP71) under an excitation wavelength of 550 nm.

### Statistical analysis

Values are expressed as the mean ± standard error of at least three independent experiments for each treatment, and Duncan’s multiple range test (P < 0.05) was used to estimate statistical significance.

## Additional Information

**How to cite this article**: Zheng, Y. *et al*. The relationship between vacuolation and initiation of PCD in rice (*Oryza sativa*) aleurone cells. *Sci. Rep.* **7**, 41245; doi: 10.1038/srep41245 (2017).

**Publisher's note:** Springer Nature remains neutral with regard to jurisdictional claims in published maps and institutional affiliations.

## Supplementary Material

Supplementary Information
