# Supplementary material for: The relationship between vacuolation and initiation of PCD in rice (Oryza sativa) aleurone cells
Source: Sci Rep. 2017 Jan 24;7:41245. doi: 10.1038/srep41245 (PMC5259747; doi:10.1038/srep41245)
Supplement: Supplementary Information [file srep41245-s1.pdf]

Supplementary Figures

Title:

The relationship between vacuolation and initiation of PCD in rice (*Oryza sativa*) aleurone cells

Authors:

Yan Zheng, Heting Zhang, Xiaojiang Deng, Jing Liu and Huiping Chen

Figure S1 Zheng *et al.*

Bright field	
Fluorescence	

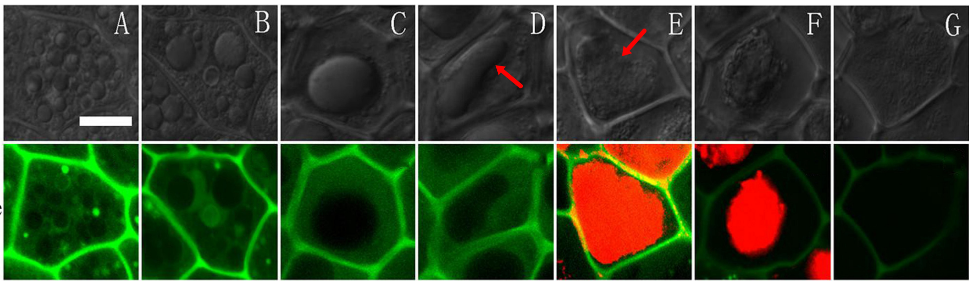

Figure S1. Morphological changes of vacuoles in the PCD of rice aleurone layers. The layers stripped from the seeds imbibed for 2 d were respectively incubated in distilled water for another 5 d. The viability of cells was determined by staining with FDA and FM4-64. Live cells emit green fluorescence, and dead cells red. Morphology of vacuoles was observed by LSCM at the indicated time. (A-D) Since fluorescein is excluded from vacuoles, the cytosol appear FDA green fluorescence, while the vacuoles appears bark against a bright background. And Corresponding images of bright field show the intact of the vacuoles. (E-G) The dead cells with shrinkage protoplasm emit FM4-64 red fluorescence, and the dead cell without protoplasm gives no fluorescence. After the large central vacuole elongates and deforms (D, arrow), the protoplasm appear FM4-64 red fluorescence, and the protoplasm separate from the wall (E, arrow). Finally, the protoplasm shrinking into a mass appear FM4-64 red fluorescence (F), and the cavity of the dead cell does not appear fluorescence (G). In addition, the green fluorescence of cells wall is autofluorescence.

Figure S2 Zheng *et al.*

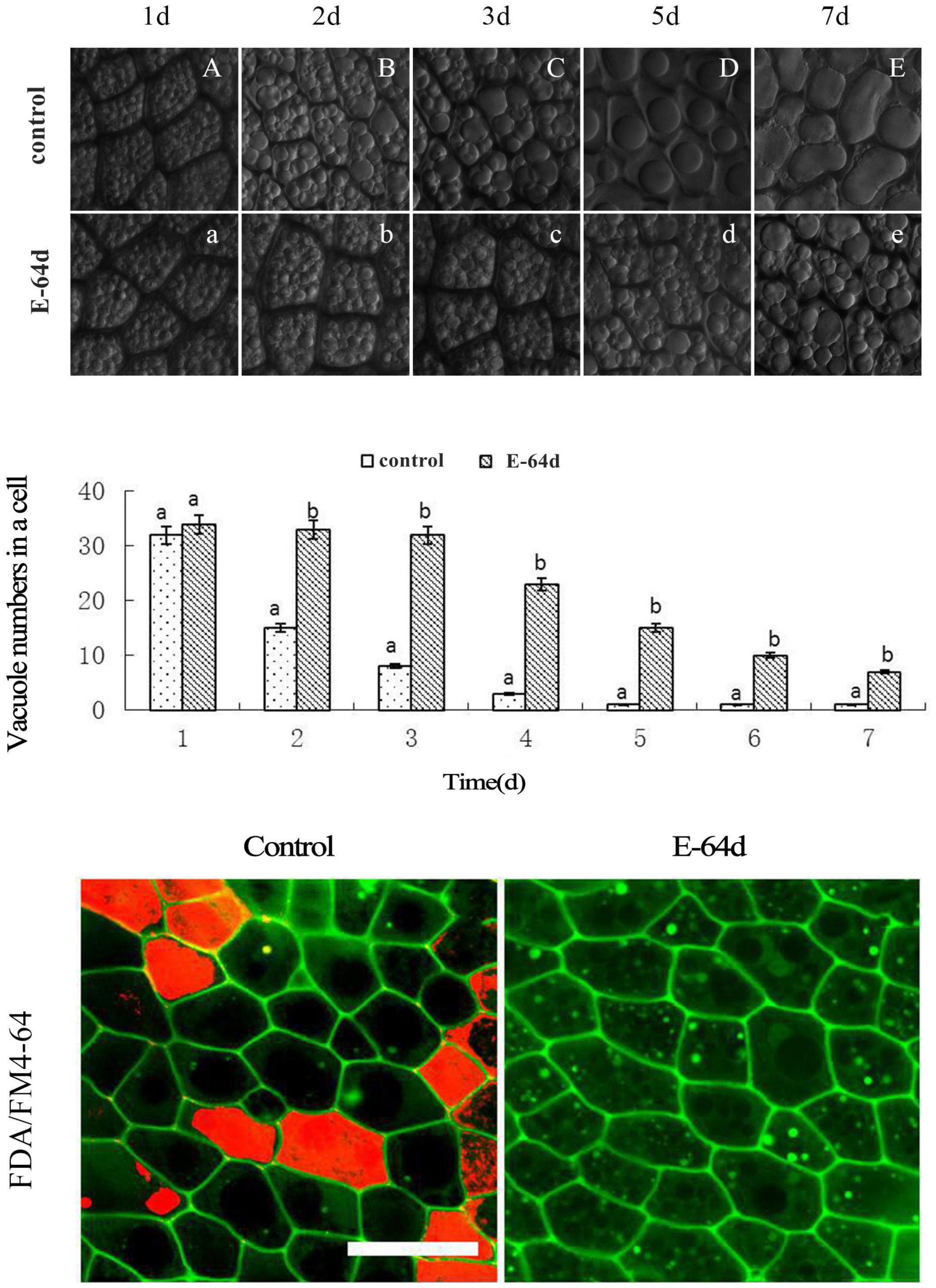

Figure S2. Effects of E-64d on the vacuolation and PCD in rice aleurone layers. The layers were respectively stripped from the seeds incubated in distilled water

(control) and 1 μg L^{-1} E-64d (vacuole fusion inhibitor) for 7 d. (A) The vacuolation of layers incubated at 1 d, 2 d, 3 d, 5 d and 7 d were observed by LSCM. The bar represents 20 μm. (B) The number of vacuoles in a cell was counted from 1 d to 7 d. (C) The layers incubated for 7 d were stained by FDA and FM 4-64, and observed by LSCM. Live cells emit FDA green fluorescence, and dead cells emit FM4-64 red fluorescence. The bar represents 50 μm. The assay was performed at least three times and a representative aleurone layer section is shown. Data are means ± SD of at least four independent measurements from different experiments, and different letters indicate a significant different at $p < 0.05$ according to Duncan's multiple range test.
